# Supplementary material for: Structural Heterogeneity of the Rabies Virus Virion
Source: Viruses. 2024 Sep 11;16(9):1447. doi: 10.3390/v16091447 (PMC11437398; doi:10.3390/v16091447)
Supplement: Supplementary file 1 [file viruses-16-01447-s001.zip › viruses-3189040-supplementary.pdf]

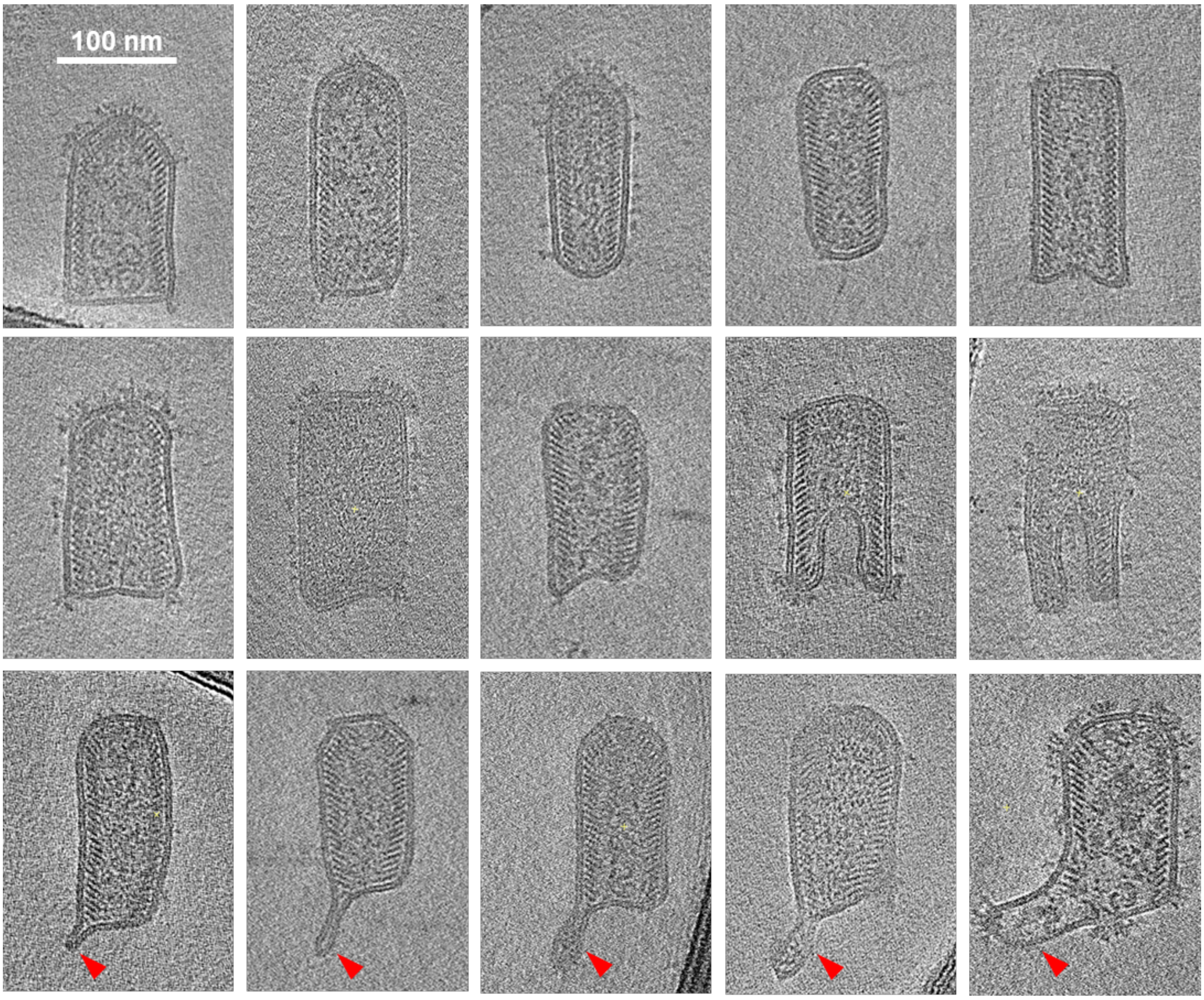

Supplementary Figure S1. RABV virions show morphological variability. Gallery of density slices from reconstructed tomograms of RABV particles depicting structural heterogeneity and showing differences in the shape of the tip (i.e., conical, dome-like or flattened) and the base (i.e., flattened, with a convex or concave curvature, or with a pronounced invagination), and the presence or absence of a tail-like appendage of variable dimensions at the base (red arrowhead in the panels of the bottom row).

A

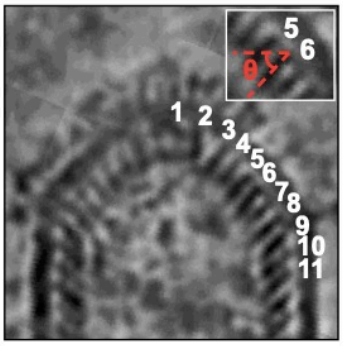

B

Turn #	Horizontal angle θ
1	88°
2	84°
3	53°
4	51°
5	47°
6	45°
7	44°
8	43°
9	41°
10	41°
11	41°

C

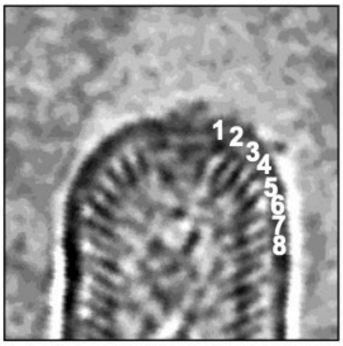

D

Turn #	Horizontal angle θ
1	53°
2	50°
3	49°
4	47°
5	45°
6	31°
7	31°
8	31°

E

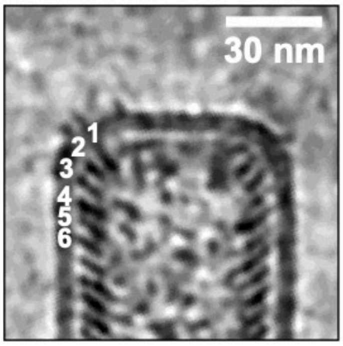

F

Turn #	Horizontal angle θ
1	53°
2	50°
3	44°
4	37°
5	37°
6	37°

Supplementary Figure S2. Three types of RABV tips. Representative conical (**A**), dome-like (**C**) and flat-topped tips (**E**) of RABV are shown in slices from reconstructed tomograms. The turn layers of the helical RNP are annotated. Inset: zoom-in view of layers 5 and 6 in (**A**), with red dashed lines indicating the inclination (horizontal angle θ) of M-N subunits. The scale bar corresponds to 30 nm. (**B**, **D**, **F**) Horizontal angles of different helical turn layers in the tips in (**A**), (**C**) and (**E**).

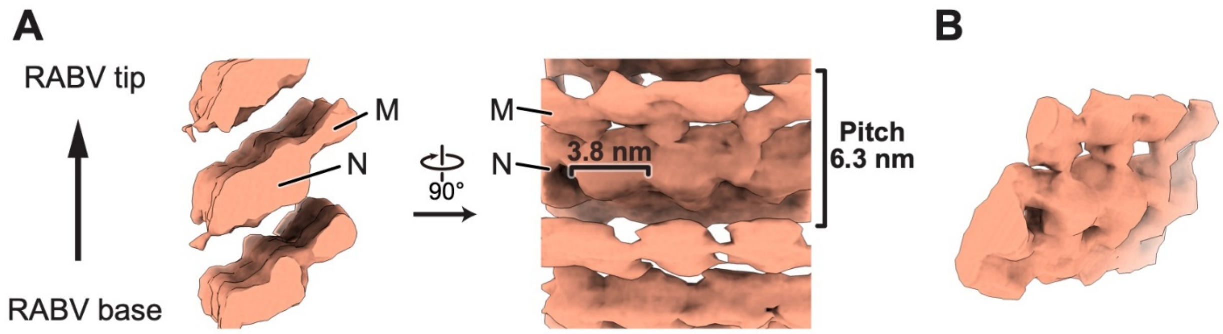

Supplementary Figure S3. Subtomogram-averaged structure of RABV M-N used for placing back the subtomogram average of M-N into the tomograms to measure the pitch in the RABV trunk. (**A**) The subtomogram-averaged structure was aligned from subtomograms evenly distributed along the virion trunk at different heights, revealing the average pitch of the virions. The arrow on the left indicates the directionality of the virion. The pitch of RNP helix and the distance between subsequent units in one turn are indicated. (**B**) One layer of the subtomogram-averaged M-N used to be placed back into the reconstructed tomograms.

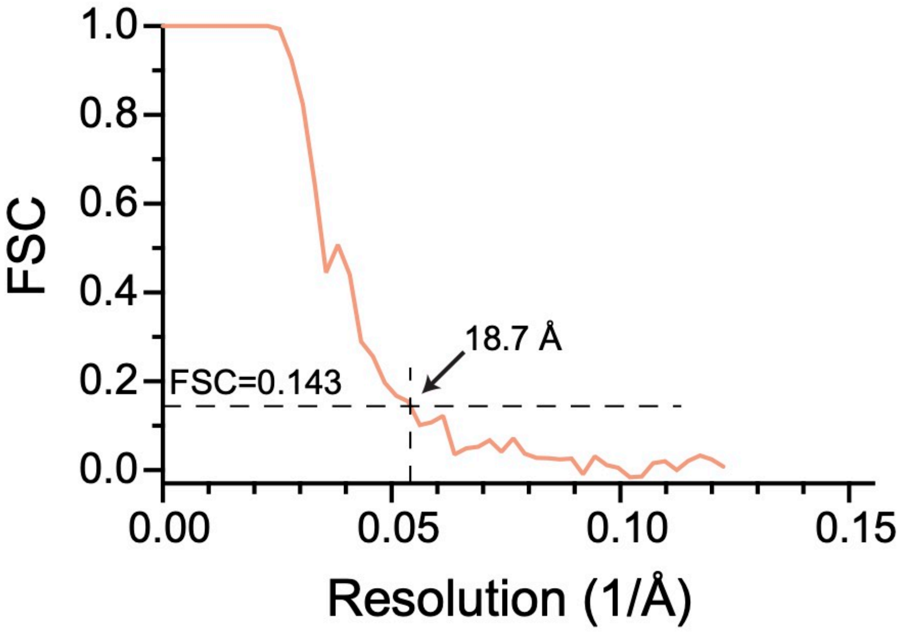

Supplementary Figure S4. Plot of the Fourier shell correlation (FSC) as a function of the spatial frequency demonstrating the resolutions of final reconstructions of the subtomogram average of RABV M-N.

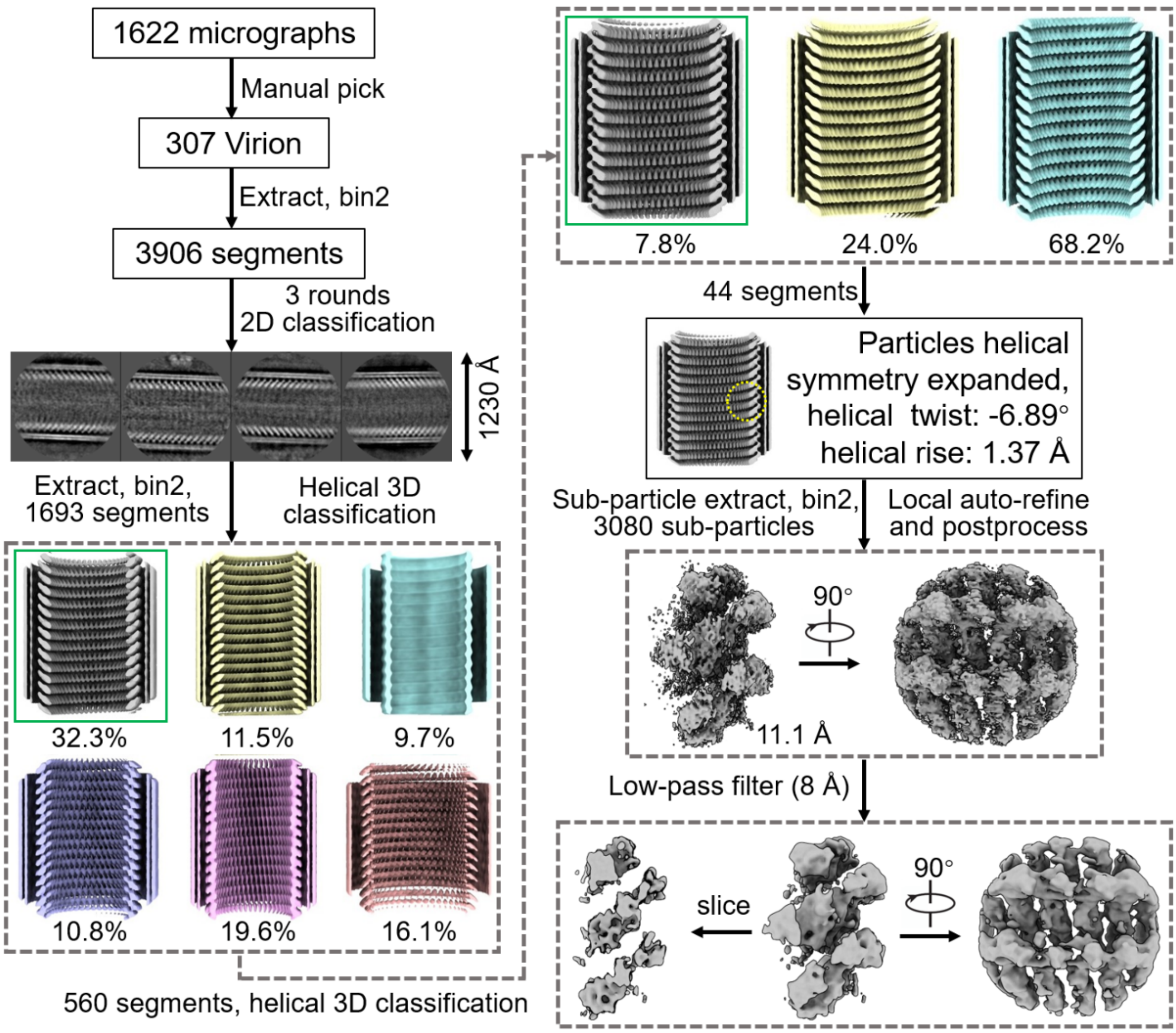

Supplementary Figure S5. Data processing workflow for helical sub-particle reconstruction.

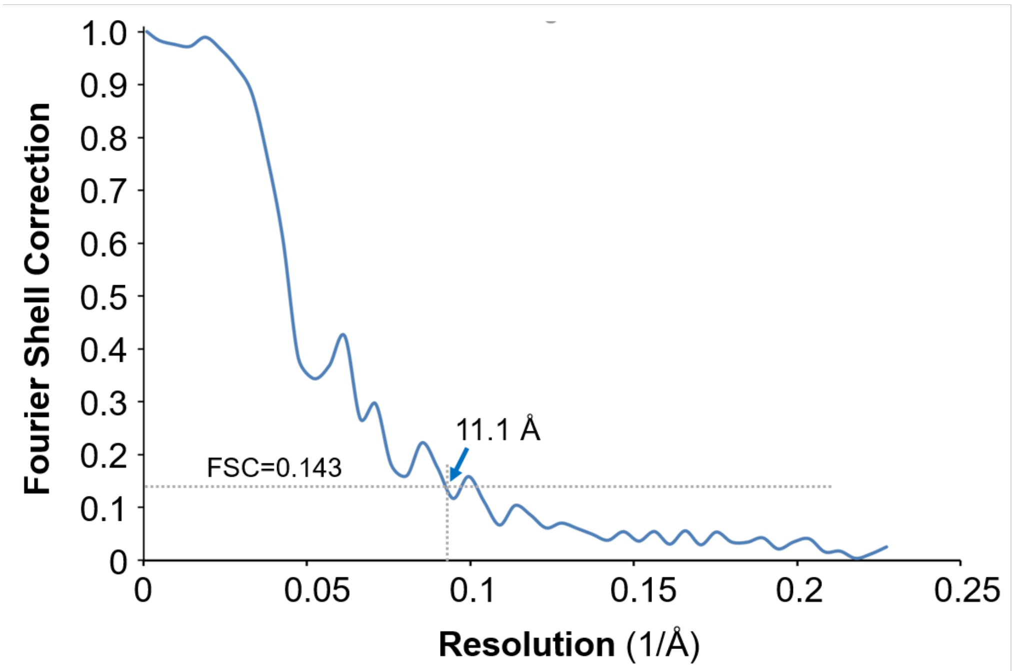

Supplementary Figure S6. Global resolution evaluation based on “gold-standard” Fourier shell correction (FSC) coefficient as a function of spatial frequency generated by RELION, showing a resolution of 11.1 Å based on the 0.143 cut-off of FSC coefficient.

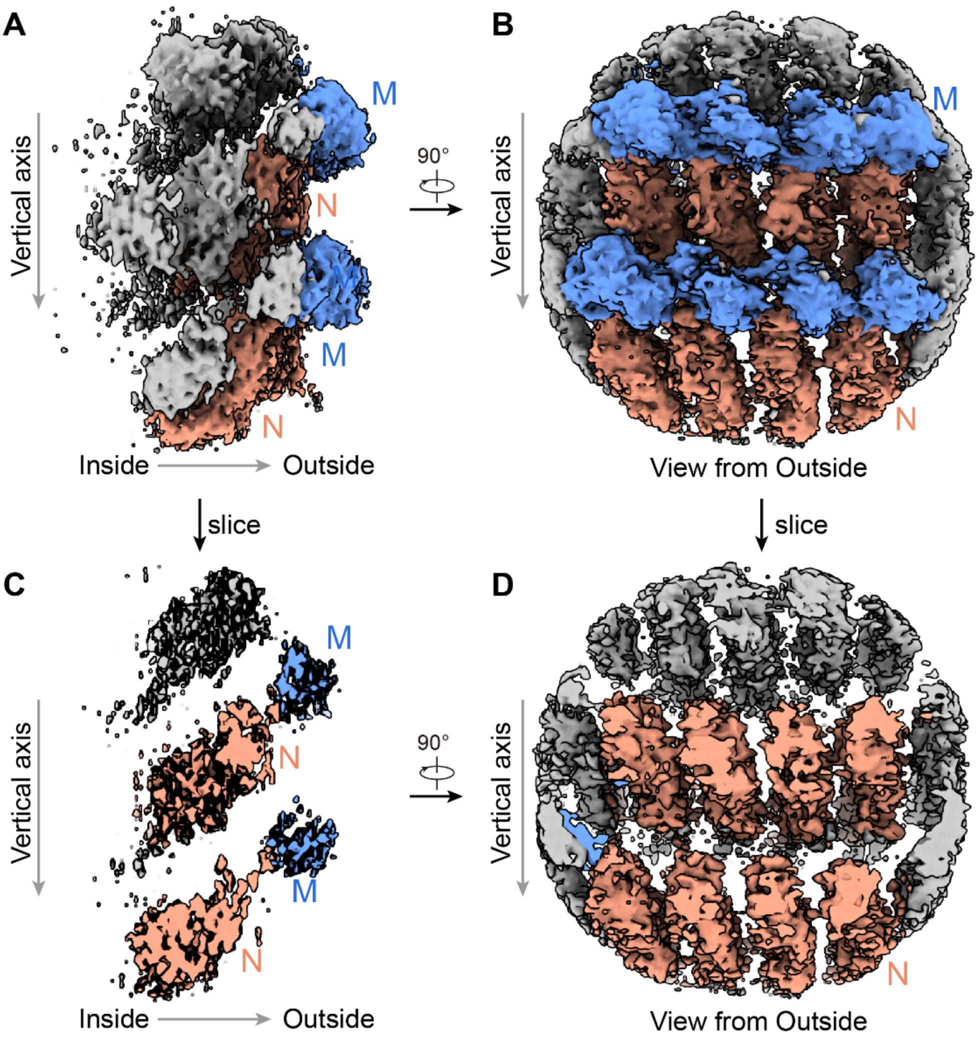

Supplementary Figure S7. CryoEM map of RABV trunk without low-pass filter. (**A** and **B**) CryoEM density maps of partial RABV nucleocapsid shown in two orthogonal views. The cryoEM map was raw without low-pass filter. Only intact N and intact M are colored for clarity, other densities are gray. N subunits are colored in orange, and M in blue. (**C** and **D**) Cross sections from **A** and **B**, respectively.

Supplementary Table S1. CryoET data collection and processing statistics

	**RABV M-N** (EMD-46612)
**Data collection**	
Microscope	Titan Krios
Magnification	×64,000
Voltage (kV)	300
Total electron exposure ($e^{-}/Å^{2}$)	120
Slit width (eV)	20
Detector	K3
Defocus range (μm)	-2.5 to -4.5
Pixel size (Å)	1.38
Software	SerialEM
Tilt-series range	±60°
Tilt-series increment	±3°
Tilt-series scheme	Bi-directional
Tilt-series used	10
**Data processing**	
Software: tilt-series alignment	IMOD
Software: denoising and missing-wedge correction	IsoNet
Software: particle picking	TomoNet
Software: final reconstruction	Relion4.0
Initial segments (no.)	7,534
Final segments (no.)	2,300
Final Box-size (px)	96
Pixel size final reconstruction (Å)	4.14
Symmetry imposed	C1
Map resolution (Å)	18.7
FSC threshold	0.143

Supplementary Table S2. CryoEM data collection and processing statistics

	**RABV virion trunk** (EMD-46621)
**Data collection and processing**	
Microscope	Titan Krios
Magnification	×81,000
Voltage (kV)	300
Exposure time (s)	1.6
Frames (no.)	40
Electron exposure (e–/Å^2)	50
Defocus range (μm)	-1.8 to -2.1
Pixel size (Å)	1.1
**Helical reconstruction**	
Initial segments extracted (no.)	3,906
Segments after Class2D (no.)	1,693
Segments after Class3D (no.)	44
Symmetry imposed	*C1*
Helical twist (°)	-6.89
Helical rise (Å)	1.37
**Sub-particle reconstruction**	
Final particles (no.)	3,080
Symmetry imposed	*C1*
Map resolution (Å)	11.1
FSC threshold	0.143
Map sharpening *B* factor (Å^2)	-136.8
Map pixel size (Å)	2.2
